# Supplementary material for: Assessment of the Effectiveness of Zone 1-Landing Hybrid TEVAR by Comparing Its Outcomes with Those of Zone 2-Landing Hybrid TEVAR
Source: J Clin Med. 2023 Aug 16;12(16):5326. doi: 10.3390/jcm12165326 (PMC10455504; doi:10.3390/jcm12165326)
Supplement: Supplementary file 1 [file jcm-12-05326-s001.zip › jcm-2516736-supplementary.pdf]

Supplementary Figure S1A

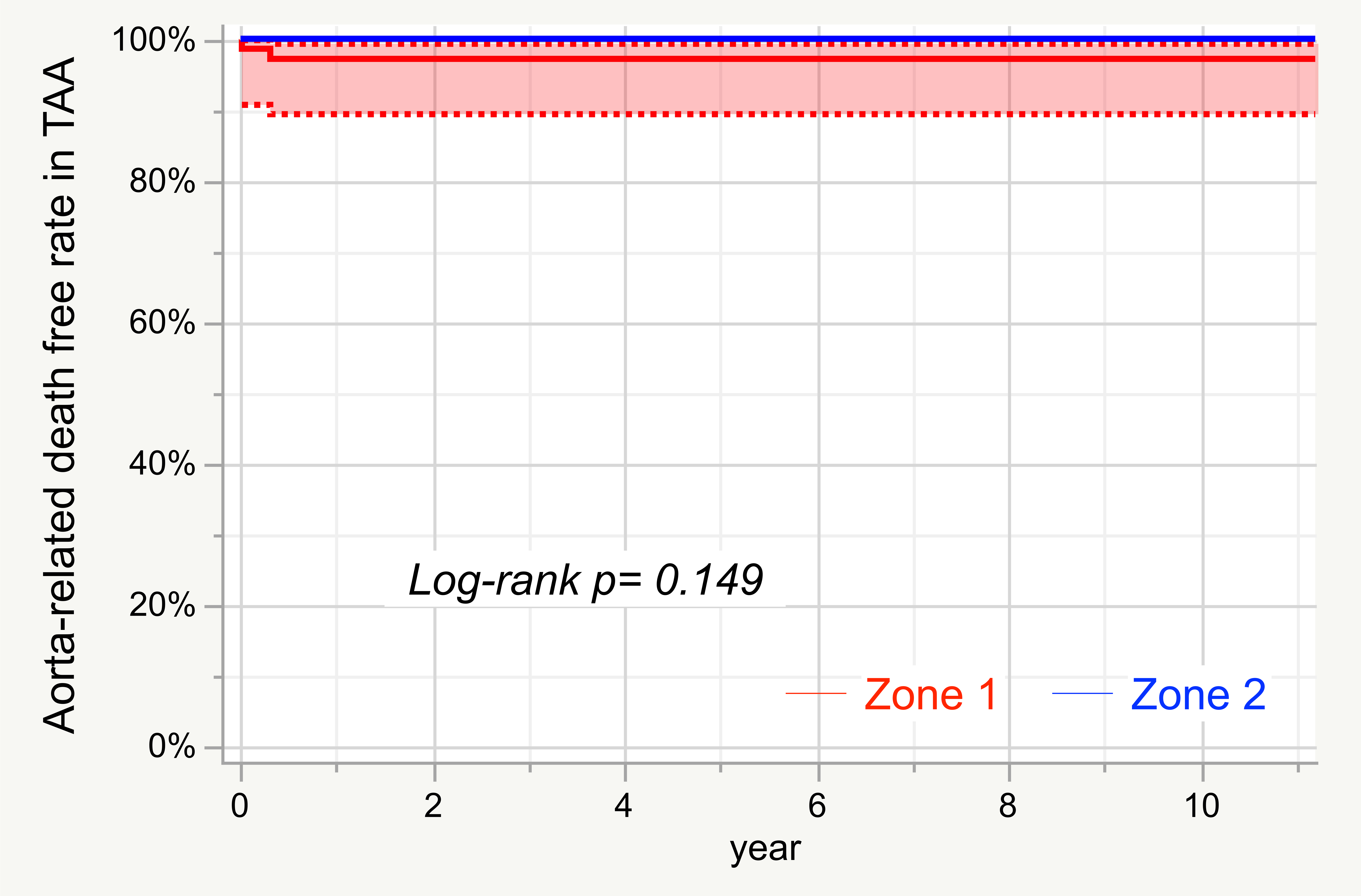

Patient at risk (n)	0	1	3	5	10
Zone 1	71	66	50	43	10
Zone 2	73	73	55	46	18

Supplementary Figure S1B

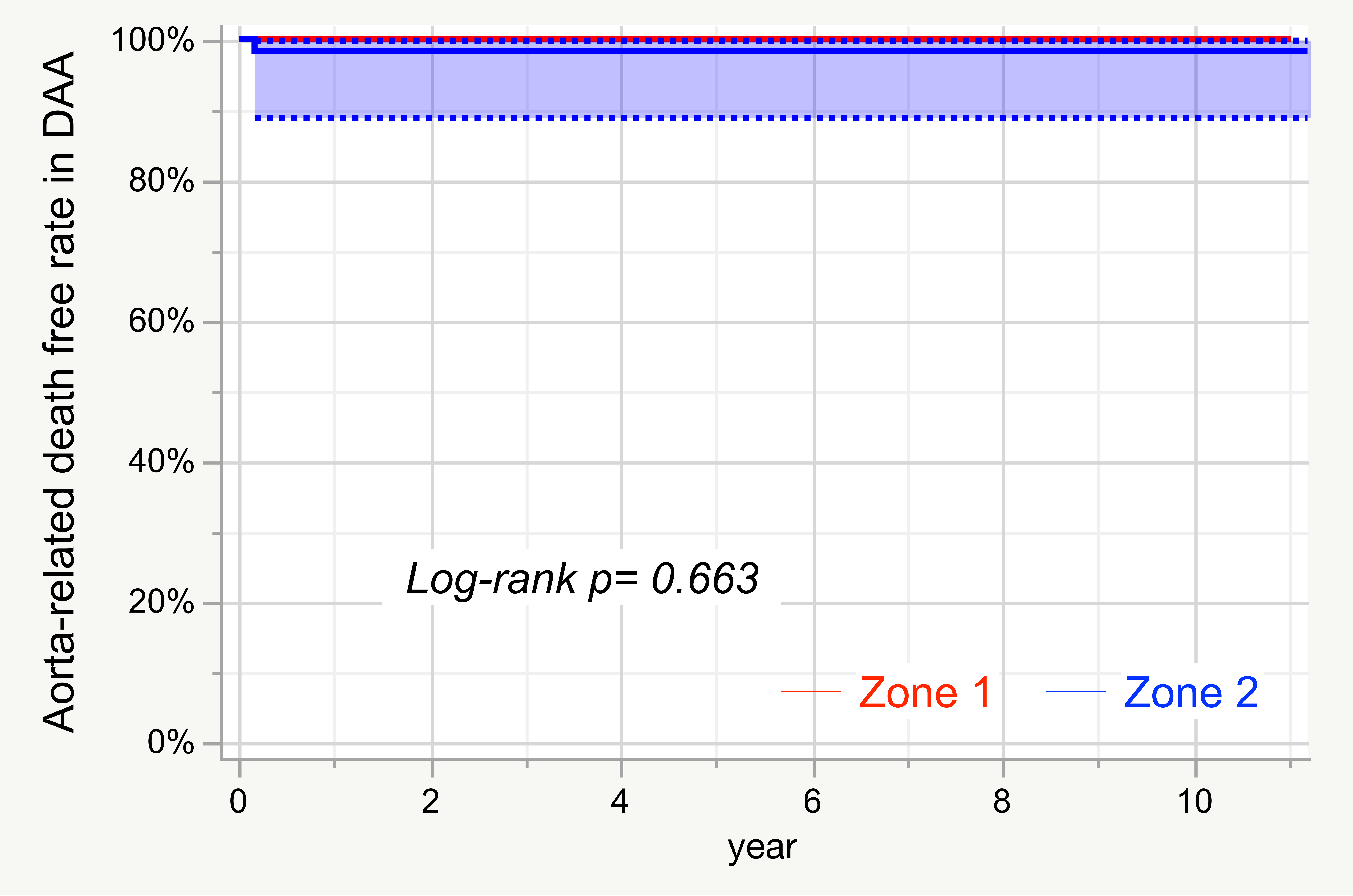

Patient at risk (n)	0	1	3	5	10
Zone 1	11	11	9	7	4
Zone 2	58	55	45	35	15

Supplementary Figure S1C

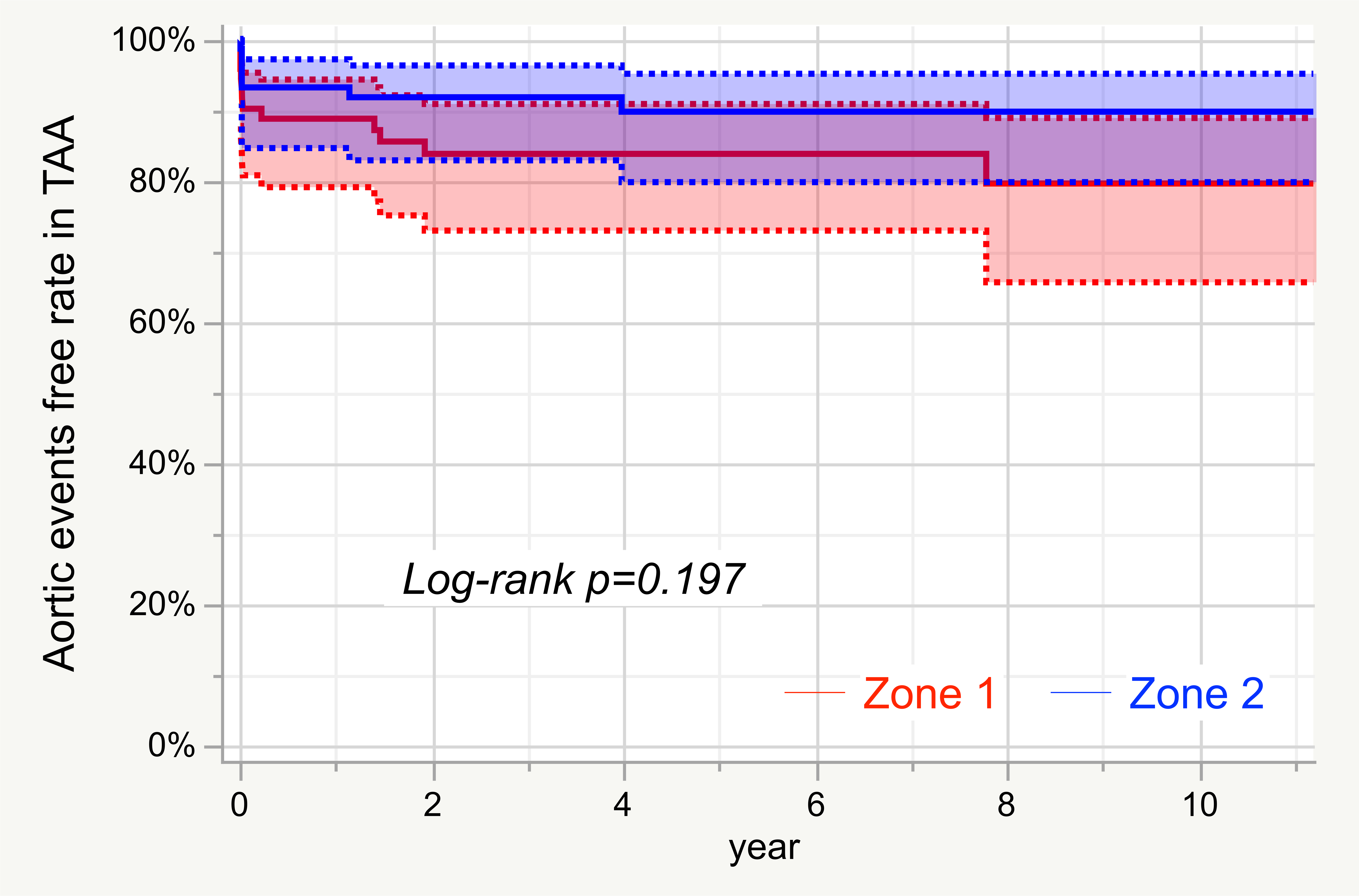

Patient at risk (n)	0	1	3	5	10
Zone 1	71	59	41	33	8
Zone 2	73	68	49	40	16

Supplementary Figure S1D

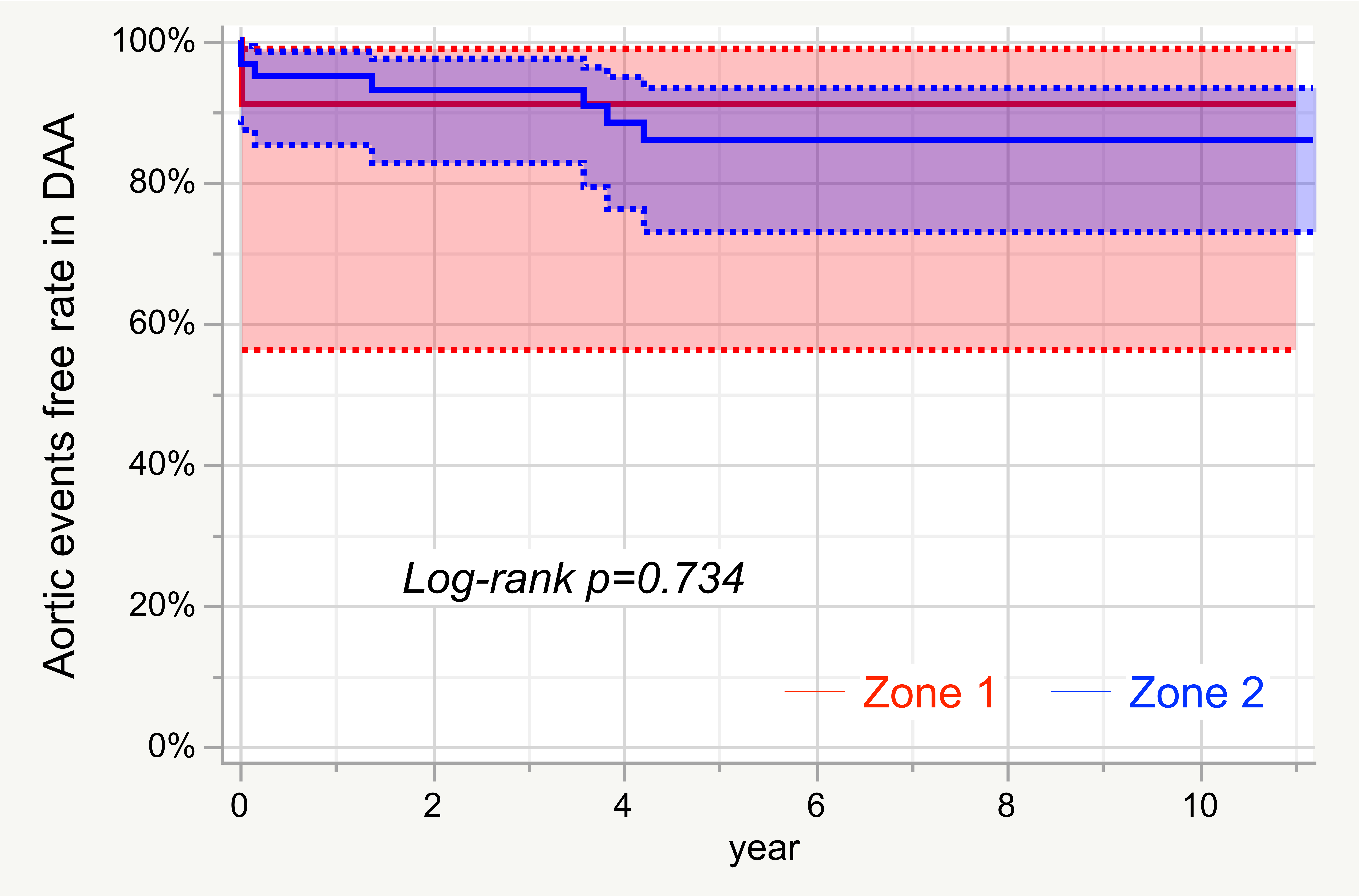

Patient at risk (n)	0	1	3	5	10
Zone 1	11	11	10	9	4
Zone 2	58	53	42	30	12

Table S1: Comparison of the outcomes in aortic pathologies

	TAA (n=144)				DAA (n=69)		
	Zone 1 n=71 (86.6%)	Zone 2 n=73 (55.7%)	*P* value		Zone 1 n=11 (13.4%)	Zone 2 n=58 (44.3%)	*P* value
**Patient characteristics**							
Age (years)	76 (69-80)	74 (67-80)	0.2518		61 (59-68)	68 (56-73)	0.1785
Age ≥80 years, n (%)	19 (26.8)	19 (26.0)	0.9205		0	5 (8.6)	0.5847
Female, n (%)	11 (15.5)	16 (21.9)	0.3234		2 (18.2)	13 (22.4)	1.00
Emergency, n (%)	7 (9.9)	5 (6.9)	0.5135		4 (36.4)	17 (29.3)	0.7248
Logistic EuroSCORE (%)	24.7±16.8	19.8±13.8	0.0596		14.4±6.0	18.7±10.5	0.1934
**Operative outcomes**							
Operative time (minutes)	191 (164-218)	139 (108-174)	<0.0001		202 (148-225)	144 (117-170)	0.0256
**In-hospital outcomes**							
30-day mortality, n (%)	1 (1.4)*	0	0.4931		0	0	1.00
Hospital mortality, n (%)	1 (1.4)*	0	0.4931		0	1 (1.7)+	1.00
Hospital stay (days)	11 (9-19)	9 (8-13)	0.0039		16 (10-22)	10 (8-16)	0.0889
Discharge at home, n (%)	67 (94.4)	71 (97.3)	0.3849		0	4 (6.9)	1.00
Complications							
Stroke, n (%)	1 (1.4)	1 (1.4)	1.00		0	0	1.00
Spinal cord injury, n (%)	1 (1.4)	1 (1.4)	1.00		0	0	1.00
Abdominal embolic event, n (%)	1 (1.4)*	0	0.4931		0	0	1.00
Aortic rupture, n (%)	0	0	1.00		0	1 (1.7)+	1.00
Endoleaks							
Type 1a, n (%)	1 (1.4)	1 (1.4)	1.00		0	1 (1.7)+	1.00
Type 1b, n (%)	1 (1.4)	0	0.4931		0	0	1.00
Type 1c, n (%)	2 (2.8)	3 (4.1)	1.00		1 (9.1)	1 (1.7)	0.2954
**Late outcomes**							
Late death, n (%)	17 (23.9)	15 (20.1)	0.6907		1 (9.1)	4 (6.9)	1.00
Complications							
RTAD, n (%)	1 (1.4)	0	0.4931		0	0	1.00
Distal SINE, n (%)	0	0	1.00		0	3 (5.2)	1.00
Stent graft infection, n (%)	1 (1.4)	0	0.4931		0	0	1.00
Bypass graft occlusion, n (%)	0	1 (1.4)	1.00		0	0	1.00
Endoleaks							
Type 1a, n (%)	1 (1.4)	1 (1.4)	1.00		0	2 (3.5)	1.00
Type 1b, n (%)	2 (2.8)	0	0.2414		0	0	1.00

Data are represented as mean ± standard deviation and median (IQR: interquartile range)
TAA: thoracic aortic aneurysm; DAA: dissecting aortic aneurysm; RTAD: retrograde type A dissection; SINE: stent graft-induced new entry; *: same patient; $^{+}$: same patient
